# Risk factors for gastrointestinal bleeding in children with Henoch-Schönlein purpura

**DOI:** 10.3389/fped.2025.1587535

**Published:** 2025-04-23

**Authors:** Dequan Su, Mi Yang, Xiaoqin Wang, Guangbo Li, Shaoxian Hong

**Affiliations:** ^1^ Department of Nephrology, Xiamen Hospital, Affiliated Children’s Hospital of Fudan University (Xiamen Children’s Hospital), Xiamen, China; ^2^ Department of Immunology and Rheumatology, Fudan University Affiliated Children’s Hospital Xiamen Hospital (Xiamen Children’s Hospital), Xiamen, China; ^3^ Department of Pediatric Intensive Care Medicine, Fudan University Affiliated Children’s Hospital Xiamen Hospital (Xiamen Children’s Hospital), Xiamen, China

**Keywords:** abdominal pain, D-dimer, gastrointestinal bleeding, Henoch-Schönlein purpura, procalcitonin, risk factors

## Abstract

**Objective:**

To analyze the risk factors associated with gastrointestinal bleeding in pediatric patients with Henoch-Schönlein purpura (HSP), with the goal of enhancing early diagnosis and treatment, preventing complications, and improving patient outcomes.

**Methods:**

The study involved 23 pediatric patients with HSP who experienced gastrointestinal bleeding, forming the study group. They were admitted to our hospital from June 2023 to June 2024. For comparison, a control group composed of 44 children with HSP but without gastrointestinal bleeding, admitted during the same timeframe, was established. Data on clinical characteristics, laboratory results, and imaging findings were collected. Both univariate and multivariate analyses were conducted to identify potential risk factors.

**Results:**

A total of 67 children with HSP were divided into two groups: those with gastrointestinal bleeding (23 cases) and those without (44 cases). The occurrence of abdominal pain and incidence of intestinal wall thickening detected by gastrointestinal ultrasonography were significantly higher in the group with gastrointestinal bleeding compared to the group without bleeding (*P* < 0.05). Univariate analysis revealed significant differences in eight laboratory parameters (WBC, NE, NLR, PCT, CRP, D-dimer, Fib, TT), all statistically significant (*P* < 0.05). Multivariate analysis identified three independent risk factors for gastrointestinal bleeding in children with HSP: abdominal pain (OR = 2.334, 95% CI: 0.458–11.886, *P* = 0.010), a PCT level above 0.12 ng/ml (OR = 10.010, 95% CI: 1.208–82.929, *P* = 0.033), and a D-dimer level exceeding 1.87 mg/L (OR = 3.407, 95% CI: 1.022–17.473, *P* < 0.001).

**Conclusion:**

The findings confirmed that abdominal pain, elevated PCT levels, and increased D-dimer are significant independent risk factors for gastrointestinal bleeding in pediatric patients with Henoch-Schönlein purpura. Clinicians should monitor these indicators attentively to enhance patient management and outcomes.

## 1 Introduction

Henoch-Schönlein purpura (HSP), also referred to as IgA vasculitis, represents the most prevalent form of vasculitis in children, characterized by the deposition of IgA-dominant immune complexes affecting multiple systems (1–4). The annual incidence of HSP in individuals under 18 years of age ranges from 10 to 20 per 100,000 population (5). The deposition of IgA immune complexes in the skin leads to palpable purpura and petechiae, primarily appearing on the lower extremities and buttocks, although the trunk and upper limbs can also be involved. In the gastrointestinal tract, these deposits can cause bleeding, and in the kidneys, they may lead to conditions ranging from mild proliferative glomerulonephritis to severe crescentic glomerulonephritis (6). Gastrointestinal symptoms typically include abdominal pain, hematemesis, and melena. Severe complications such as intussusception, bowel perforation, necrosis, and significant hemorrhage may develop, posing major mortality risks during the acute phase of the disease (7). The short-term prognosis of HSP largely depends on the severity of the initial gastrointestinal symptoms, whereas the long-term outlook is affected by the degree of renal impairment (7). Unaddressed or inadequately managed gastrointestinal bleeding can escalate to critical complications and potentially fatal outcomes (8). Therefore, early recognition, diagnosis, and treatment of gastrointestinal bleeding are essential to enhance patient outcomes.Despite this, effective indicators for evaluating and predicting gastrointestinal involvement in HSP remain limited in clinical practice. Platelet surface molecule expression and the release of inflammatory mediators have been implicated in inflammatory reactions (3). Recent evidence supports the efficacy of PLR in providing reliable prediction of disease conditions and prognosis in multiple conditions, including cardiovascular diseases, vasculitis, tumors, autoimmune diseases, and others (8, 9). It has emerged as a potential sensitive marker of inflammatory reactions (8, 9). However, although some studies have investigated the utility of PLR, either alone or in combination, in the assessment of HSP (9), there is still limited research on its predictive value for gastrointestinal bleeding in children with HSP.This study retrospectively examines the clinical records of children hospitalized with HSP and complicated by gastrointestinal bleeding at our facility, aiming to pinpoint contributing factors to gastrointestinal bleeding and to inform clinical practice.

## 2 Materials and methods

### 2.1 Study population

In this retrospective study, the medical records of 67 children with HSP admitted to Children's Hospital of Fudan University at Xiamen (Xiamen Children's Hospital) June 2023 to June 2024 were collected. Among them, 23 cases with gastrointestinal bleeding were included in the bleeding group, while the remaining 44 cases without gastrointestinal bleeding were included in the non-bleeding group. The study group consisted of 40 males and 27 females, resulting in a male-to-female ratio of 1.48:1. The study received approval from the Ethics Committee of Xiamen Children's Hospital, and informed consent was obtained from the patient's guardian through the signing of a consent form.

### 2.2 Inclusion criteria

#### 2.2.1 Diagnostic criteria for HSP

All participants were diagnosed with HSP according to the criteria established by EULAR/PRINTO/PRES (European League Against Rheumatism/Paediatric Rheumatology International Trials Organisation/Paediatric Rheumatology European Society) (10, 11).

#### 2.2.2 Diagnostic criteria for gastrointestinal bleeding

Patients were eligible for inclusion if they satisfied both of the following: (1) evidence of hematochezia or hematemesis; (2) children aged < 14 years; (3) children with complete clinical data

### 2.3 Exclusion criteria

Patients were omitted from the study if they met any of the following conditions: (1) Purpura due to other causes, such as idiopathic thrombocytopenic purpura; (2) Gastrointestinal bleeding due to peptic ulcers, acute appendicitis, or acute intestinal obstruction.

### 2.4 Research methods

Data recorded for the study included demographic details, length of hospital stay, and the season in which symptoms appeared for each child. The laboratory measurements taken at the admission were: white blood cell count (WBC), neutrophil count (NE), lymphocyte count (LY), neutrophil-to-lymphocyte ratio (NLR), hemoglobin levels (HGB), platelet count (PLT), C-reactive protein (CRP), liver enzymes alanine aminotransferase (ALT) and aspartate aminotransferase (AST), serum albumin (ALB), complement components C3 and C4, prothrombin time (PT), activated partial thromboplastin time (APTT), D-dimer (D-D), and thrombin time (TT).

### 2.5 Statistical methods

Statistical analysis was conducted using SPSS version 22.0. Continuous variables that were normally distributed were presented as mea*n* ± standard deviation (mean ± SD), and comparisons between groups were made using the t-test. For variables not normally distributed, medians and interquartile ranges [M (IQR)] were reported, and comparisons between groups were made using the Mann–Whitney *U*-test. Categorical variables were reported as *n* (%), and group comparisons were made using the chi-square test. Receiver operating characteristic (ROC) curves were used to determine the area under the curve (AUC), *P*-values, and cutoff values based on the maximum Youden index, along with the sensitivity and specificity for each continuous variable. These variables were then dichotomized based on the cutoff values. Logistic regression was used for multivariate analysis, with a significance level set at *P*-value <0.05.

### 2.6 Approval and consent to participate

The studies involving human participants were reviewed and approved by Ethical committee of Children Hospital of Xiamen (XMSETYY-2024-61). Written informed consent to participate in this study was provided by the participants' legal guardian/next of kin.This is a retrospective study, clinical trial number is not applicable.

## 3 Results

### 3.1 Baseline demographic and clinical characteristics

In this study, 23 cases with gastrointestinal bleeding were included in the bleeding group, while the remaining 44 cases without gastrointestinal bleeding were included in the non-bleeding group. A comparison of demographic characteristics between the groups showed no significant differences in terms of age, gender, residence, age at disease onset, rash extent (whether it extended beyond the buttocks), presence of joint swelling and pain, or vomiting (*P* > 0.05). However, the occurrence of abdominal pain and incidence of intestinal wall thickening detected by gastrointestinal ultrasonography were significantly higher in the group with gastrointestinal bleeding compared to the group without bleeding (*P* < 0.05). Moreover, the average hospital stay was considerably longer for the gastrointestinal bleeding group (15.17 ± 8.57 days) compared to the non-bleeding group (7.25 ± 2.58 days), as a a result of gastrointestinal bleeding (Table 1).

**Table 1:** Comparison of baseline data between the two groups.

Clinical characteristics	Gastrointestinal bleeding group (*n* = 23), (%)	Non-gastrointestinal bleeding group (*n* = 44), (%)	*P*-value
Age (years)	7.00 ± 3.25	6.91 ± 3.03	0.936
Residence			0.587
Urban	17 (25.37)	29 (43.28)	
Rural and suburban	6 (8.96)	15 (22.39)	
Gender			0.345
Male	15 (22.39)	25 (37.31)	
Female	8 (11.94)	19 (28.36)	
Season of onset			0.562
Spring	8 (11.94)	14 (20.90)	
Summer	3 (4.48)	6 (8.96)	
Autumn	2 (2.99)	4 (5.97)	
Winter	10 (14.93)	20 (29.85)	
Rash distribution			
Lower limbs and buttocks	17 (25.37)	35 (52.24)	0.408
Beyond lower limbs and buttocks	6 (8.96)	9 (13.43)	
Joint swelling and pain			0.072
Yes	8 (11.94)	25 (37.31)	
No	15 (22.39)	19 (28.36)	
Abdominal pain			0.001
Yes	21 (31.34)	23 (34.33)	
No	2 (2.99)	21 (31.34)	
Vomiting			
Yes	14 (20.90)	19 (28.36)	0.089
No	9 (37.31)	25 (37.31)	
Gastrointestinal ultrasound			<0.001
Intestinal wall thickening	21 (31.34)	13 (19.40)	
Normal	2 (2.99)	31 (46.27)	
Length of Hospital Stay (days)			
	15.17 ± 8.57	7.25 ± 2.58	<0.001

### 3.2 Comparison of laboratory parameters between the two groups

We compared laboratory parameters such as WBC, NE, LY, NLR, CRP, PCT, AST, ALT, IgG, IgA, C3, C4, Fib, D-dimer, APTT, and TT between children with gastrointestinal bleeding and those without. We found significant differences in WBC, NE, NLR, CRP, PCT, Fib, D-dimer, and TT (*P* < 0.05). There were no statistically significant differences in the other parameters (*P* > 0.05) (Table 2).

**Table 2:** Comparison of laboratory parameters between the two groups.

Laboratory parameter	Gastrointestinal bleeding group (*n* = 23)	Non-gastrointestinal bleeding group (*n* = 44)	*t*	*P*-value
WBC (*10^−9^/L)	17.46 ± 8.70	12.79 ± 6.30	2.493	0.047
NE (*10^−9^/L)	13.14 ± 7.86	9.21 ± 5.78	2.315	0.045
LY (*10^−9^/L)	2.96 ± 1.32	2.88 ± 1.22	0.234	0.571
NLR	5.28 ± 0.70	3.84 ± 0.45	3.028	0.018
CRP (mg/L)	19.04 ± 4.28	6.55 ± 1.29	3.485	<0.001
HGB (g/L)	135.13 ± 13.79	128.04 ± 11.10	2.267	0.829
PCT (ng/ml)	0.94 ± 0.62	0.12 ± 0.02	1.866	0.001
AST (U/L)	20.03 ± 2.58	29.75 ± 7.98	−0.874	0.391
ALT (U/L)	14.41 ± 1.84	22.49 ± 9.82	−0.597	0.332
IgG (g/L)	9.05 ± 3.64	12.07 ± 3.48	−3.304	0.254
IgA (g/L)	1.74 ± 0.85	2.23 ± 0.72	−2.451	0.503
C3 (g/L)	0.88 ± 0.18	1.01 ± 0.24	−2.227	0.286
C4 (g/L)	0.21 ± 0.06	0.43 ± 0.02	−1.348	0.114
Fib (g/L)	4.16 ± 0.99	9.17 ± 1.66	−2.098	<0.001
D-dimer(mg/L)	3.31 ± 0.41	2.41 ± 0.57	−0.224	0.001
FDP (ug/ml)	12.87 ± 2.38	7.11 ± 1.27	2.350	0.208
APTT (s)	29.37 ± 1.02	28.84 ± 1.30	1.786	0.103
TT (s)	14.14 ± 0.66	12.71 ± 0.85	1.140	<0.001

WBC, white blood cells; NE, neutrophil; LY, lymphocyte; NLR, ratio of neutrophil count to lymphocyte count; CRP, C-reactive protein; HGB, hemoglobin; PCT, Procalcitonin; AST, glutamic-pyruvic transaminase; ALT, glutamic oxaloacetic transaminase; IgG, Immunoglobulin G; IgA, immunoglobulin A; C3, complement 3; C4, complement 4; Fib, fibrinogen; FDP, fibrinogen degradation products; APTT, activated partial thromboplastin time; TT, clotting time;.

### 3.3 Determining the cutoff value of converting continuous variables to dichotomous variables in the training set

In univariate analysis, seven continuous variables (WBC, NE, NLR, PCT, CRP, Fib, D-dimer, and TT) showed statistically significant differences between the gastrointestinal bleeding and non-gastrointestinal bleeding groups (*P* < 0.05). For ease of clinical application, Receiver Operating Characteristic (ROC) curves were employed to determine the area under the curve (AUC), *P*-values, and the optimal cutoff values based on the maximum Youden index for each variable, including their sensitivity and specificity. These established cutoff values were then applied to convert the continuous variables into dichotomous variables (Table 3).

**Table 3:** Cutoff values of converting continuous variables to dichotomous variables in the training set.

Cut-off value	AUC (95% CI)	*P*-value	Sensitivity	Specificity
WBC > 11.22*10^−9^/L)	0.683 (0.550–0.816)	0.015	0.783	0.512
CRP (>17.38 mg/dl)	0.716 (0.585–0.848)	0.004	0.478	0.860
NE (>9.50*10^−9^/L)	0.667 (0.532–0.802)	0.026	0.565	0.711
NLR (>4.07)	0.631 (0.485–0.777)	0.041	0.609	0.651
PCT (>0.12 ng/ml)	0.638 (0.486–0.789)	0.047	0.522	0.791
Fib (<4.31 g/L)	0.603 (0.462–0.763)	0.172	0.302	0.960
D-dimer (>1.87 mg/L)	0.474 (0.335–0.612)	0.026	0.957	0.674
TT (>7.18 s)	0.539 (0.400–0.679)	0.041	0.957	0.456

AUC, area under curve; CI, confidence interval; WBC, white blood cells; NLR, ratio of neutrophil count to lymphocyte count; CRP, C-reactive protein; NE, neutrophil; PCT, procalcitonin; Fib, fibrinogen; APTT, activated partial thromboplastin time; TT, clotting time; s, seconds.

### 3.4 Logistic multivariate analysis of gastrointestinal bleeding in HSP

We incorporated eight laboratory measures (WBC, NE, NLR, PCT, CRP, Fib, D-dimer, and TT) and two clinical symptoms (abdominal pain and gastrointestinal wall thickening) into a binary logistic regression model for further multivariate analysis. This analysis identified that initial abdominal pain (OR = 2.334, 95% CI: 0.458–11.886, *P* = 0.010), a PCT level above 0.12 ng/ml (OR = 10.010, 95% CI: 1.208–82.929, *P* = 0.033), and a D-dimer level exceeding 1.87 mg/L (OR = 3.407, 95% CI: 1.022–17.473, *P* < 0.001) independently increase the risk of gastrointestinal bleeding in children diagnosed with HSP. We conducted a power analysis on the independent risk factors for GI (PCT and D-dimer levels), and their power values were both 1, indicating reliable conclusions (Table 4).

**Table 4:** Multivariate analysis of predictive factors for gastrointestinal bleeding in abdominal HSP patients.

Clinical characteristics/Laboratory parameter	B	S.E.	Wald	*P*-value	OR	95% CI
Abdominal pain	2.796	1.080	6.700	0.010	2.334	0.458–11.886
Ultrasound-indicated intestinal wall thickening	2.335	1.461	2.555	0.110	10.330	0.590–136.040
WBC(>11.22*10^−9^/L)	1.935	1.004	3.718	0.054	6.927	0.969–49.541
NLR (>4.07)	0.807	0.831	1.041	0.308	2.334	0.458–11.886
CRP (>17.38 mg/dl)	0.897	1.131	0.629	0.428	2.452	0.267–22.515
NE (>9.50*10^−9^/L)	0.944	1.005	0.883	0.347	2.571	0.359–18.424
PCT (>0.12 ng/ml)	2.304	1.079	4.560	0.033	10.010	1.208–82.929
FiB (<4.31 g/L)	2.577	1.224	0.823	0.561	0.561	0.051–6.176
D-dimer (>1.87 mg/L)	2.898	1.484	7.366	<0.001	3.407	1.022–17.473
TT (>7.18 s)	−0.793	1.578	0.252	0.616	0.453	0.021–9.981

CI, confidence interval; WBC, white blood cells; NLR, ratio of neutrophil count to lymphocyte count; CRP, C-reactive protein; NE, neutrophil; PCT, Procalcitonin; Fib, fibrinogen; TT, clotting time; s, seconds.

## 4 Discussion

In our study, we evaluated the clinical symptoms and laboratory indicators of 23 children with HSP who experienced gastrointestinal bleeding. We identified abdominal pain, PCT levels above 0.12 ng/ml, and D-dimer levels exceeding 1.87 mg/L as independent predictors of gastrointestinal bleeding in children with HSP, underscoring the importance for clinicians to monitor these markers closely.

Our findings revealed that abdominal pain is a risk factor for gastrointestinal bleeding in children with HSP in both univariate and multivariate analyses, which aligns with previous studies (11, 12). One study reported that that children who initially presented with abdominal pain were 4.5 times more likely to experience gastrointestinal bleeding compared to those whose initial symptom was a rash (11). Furthermore, another study reported that all children with HSP who developed gastrointestinal bleeding had experienced abdominal pain (13). Similarly, in our analysis, 91.3% of the patients in the gastrointestinal bleeding group presented with abdominal pain, aligning with these earlier findings. The mechanism linking abdominal pain to gastrointestinal bleeding is likely due to immune dysregulation in children with HSP. During the acute phase of the condition, particularly when the abdomen is affected, diminished cellular immunity fails to adequately suppress inflammatory cytokines, leading to their overproduction. This excess triggers B lymphocyte activation and proliferation, boosting IgA production. The accumulation of IgA immune complexes in blood vessels, especially within the gastrointestinal tract's dense capillary network and the intestinal villi, instigates small-vessel vasculitis. The complement system's activation and the resultant release of inflammatory mediators induce tissue damage and, in severe instances, necrosis. The formation of platelet microthrombi aggravates localized ischemia and necrosis, causing intestinal spasms and the consequential abdominal pain (13, 14).

In this study, we observed significantly higher D-dimer levels in the gastrointestinal bleeding group of children with HSP compared to the non-gastrointestinal bleeding group, establishing it as an independent risk factor for gastrointestinal bleeding (OR = 3.407, 95% CI: 1.022–17.473, *P* < 0.001). This finding aligns with results from other studies (15, 16). One study suggested that the pathophysiology of HSP not only involves IgA immune complex-mediated endothelial damage leading to platelet adhesion and aggregation but also the activation of both intrinsic and extrinsic coagulation pathways, which contributes to a hypercoagulable state in affected children (17). Other studies have indicated that while prothrombin time (PT) and activated partial thromboplastin time (APTT) generally remain within normal limits during the onset of pediatric HSP, D-dimer levels are frequently elevated (8, 18). This may be related to the following factors: 1. D-dimer is a sensitive indicator of a prothrombotic state, whereas abnormalities in APTT and PT only become apparent in more severe hypercoagulable conditions. 2. The hypercoagulability in children with HSP is due to increased platelet aggregation, without significant changes in the functions of intrinsic and extrinsic coagulation pathways.

The occurrence of gastrointestinal bleeding in HSP is associated with multiple inflammatory factors, including procalcitonin (PCT) (19). PCT is involved in activating the complement system and boosting the phagocytic capabilities of macrophages, thus playing a role in modulating immune responses (20). Numerous studies have reported that PCT levels are notably higher in children with HSP who suffer from gastrointestinal bleeding, indicating its potential as a predictive marker for this condition (21, 22). Our study confirmed that there was a statistically significant difference in PCT levels between groups of HSP children with and without gastrointestinal bleeding, as per univariate analysis (*P* < 0.05). This indicates that children with HSP and PCT levels greater than 0.12 ng/ml are at increased risk for gastrointestinal bleeding. Additionally, multivariate logistic regression analysis showed a significant difference in PCT levels between the groups (*P* < 0.05), affirming that PCT levels above 0.12 ng/ml serve as an independent risk factor for gastrointestinal bleeding in children with HSP, in agreement with prior studies (23).

Several prior studies have established a link between increased NLR and the incidence of gastrointestinal bleeding in children with HSP (24–26). The NLR serves as an indicator of the immune system's response to ongoing inflammation, injury, or stress, where a higher ratio correlates with elevated levels of various pro-inflammatory cytokines (23). During the acute phase of HSP, pro-inflammatory cytokines such as interleukins and tumor necrosis factor are secreted, leading to an increase in the NLR (27). In a study by Fu et al. (26), which included 1,691 patients with HSP, 431 of whom had gastrointestinal complications, it was found that the NLR was significantly higher among these patients compared to the 833 without gastrointestinal issues (OR = 1.09, 95% CI: 0.62–1.57, *P* < 0.001). This indicated an association between a higher NLR and gastrointestinal manifestations in HSP patients. In our analysis, univariate analysis identified a higher NLR as a risk factor for gastrointestinal bleeding in children with HSP. However, multivariate analysis did not confirm NLR as an independent risk factor, potentially due to the limited sample size of our study. Further studies with larger sample sizes are recommended to validate these findings.

This study has several limitations. First, it is a single-center cohort study that features a small number of participants. Second, the data collection process encountered issues with missing data, which could potentially exclude them from inter-group comparisons and introduce bias into the results. These factors limit the application and reliability of our findings. Future studies should consider implementing multi-center, prospective, randomized controlled cohort studies to enhance the robustness and applicability of these results.

## 5 Conclusion

Our study found that, in children with HSP, abdominal pain, PCT > 0.12 ng/ml, and D-dimer > 1.87 mg/L are independent risk factors for gastrointestinal bleeding. These findings are crucial for clinical practitioners, as they highlight key indicators that should be vigilantly monitored and considered in both the diagnostic and treatment processes. Early identification and management of these risk factors could improve outcomes by facilitating timely and targeted therapeutic interventions, potentially reducing the severity and progression of gastrointestinal complications in children with HSP.

## Data Availability

The original contributions presented in the study are included in the article/Supplementary Material, further inquiries can be directed to the corresponding author.
